# Social influences on seeking help from mental health services, in-person and online, during adolescence and young adulthood

**DOI:** 10.1186/s12888-015-0429-6

**Published:** 2015-03-07

**Authors:** Debra J Rickwood, Kelly R Mazzer, Nic R Telford

**Affiliations:** 1 Faculty of Health, University of Canberra, Kirinari Street, Bruce, ACT 2601 Australia; 2 Headspace National Youth Mental Health Foundation National Office, 485 La Trobe Street, Melbourne, 3000 Vic Australia

**Keywords:** Help-seeking, Mental health, Family, Peer, Adolescence, Youth, Service access, Online

## Abstract

**Background:**

This study provides the first comprehensive empirical evidence of developmental changes in the social influences on seeking mental health care, both in-person and online, during the critical lifestages for mental health of adolescence and young adulthood.

**Methods:**

Main source of help-seeking influence was determined via self-report for all young people accessing youth-targeted mental health services in Australia for a first episode of care over a 12 month period during 2013. This comprised 30,839 young people who accessed in-person services and 7,155 clients of the online service.

**Results:**

Results show a major developmental shift in help-seeking influence across the age range, which varied for males and females, and a striking difference between the online and in-person service modalities. The dominant influence online, regardless of age, was the young person themself. In contrast, for in-person services, the dominant influence during adolescence was family, but this changed markedly in late adolescence to favour self-influence, with a lessor, but still substantial effect of family. The influence of friends was surprisingly low.

**Conclusions:**

To support young people with mental health problems to access mental health care, the personal connection of parents and family needs to be engaged to encourage in-person service use through better mental health literacy, particularly for adolescents. In the online environment, ways to ensure that young people themselves are guided to appropriate services are required.

## Background

The peak period of vulnerability to mental health problems is during adolescence and young adulthood. According to the US national comorbidity study, three-quarters of all mental disorders commence by 24 years of age [1]. Among 13–18 year-olds, the prevalence of mental disorder with severe impairment was 22% [2]. For those aged 16–24, a national Australian survey showed that 26% had experienced an anxiety, affective or substance use disorder in the past 12 months, and this was the highest prevalence across all age groups [3].

In marked contrast to their heightened level of need, young people are generally reluctant to seek professional health care for mental health problems. The US national comorbidity study revealed that less than one in five affected adolescents received help for anxiety, eating or substance use disorders [4]. In Switzerland, a nationally representative sample of young adults aged 16–20 years reported that only 13% who needed psychological help had consulted health care [5]. Of particular concern is the lack of help-seeking by young men, who are the least likely to seek mental health care across the lifespan. Only 13% of young men aged 16–24 years who were experiencing clinically significant symptoms had sought professional help according to the national Australian survey, which compared with one-third of the young women [3].

Seeking help is a complex process with multiple decision points and, at each point, a range of factors can accelerate or regress progress [6]. The ‘treatment gap’ in mental health care has been acknowledged for some time and is evident worldwide [7]. The gap is particularly large for adolescents and young adults, who are most in need of effective early intervention in mental health care [8]. Reviews of the factors affecting help-seeking in young people identify the major barriers as problems recognising symptoms, a preference for self-reliance, and perceived stigma and embarrassment [9,10]. Facilitators are under-researched, but the available evidence points to the important role of encouragement from others, as well as positive past experiences.

The help-seeking literature has long acknowledged the role of family and friends in seeking help for mental health problems [10]. When young people reach out with concerns for their mental health, their preference is for informal support rather than professional health care. Seeking informal help is generally a first step that precedes professional service use. The informal network of family and friends is, therefore, critical in the help-seeking process.

Developmental patterns suggest that early in adolescence both boys and girls are most likely to seek help for their mental health problems from their parents, usually their mother [11]. The younger the adolescent the more influential parents are likely to be in the help-seeking process, and parental help-seeking often takes place on behalf of the adolescent, which itself can be a challenging experience with many barriers [12].

The capacity for self-referral develops during adolescence in line with growing autonomy from parents. Even though reliance on parents declines, for most adolescents and many young adults, parents continue to play a vital role in accessing professional treatment. A study of US college students confirmed that 47% of those who had sought professional help had been encouraged to do so by their mother, whereas paternal influence was only 5% [13]. Close friends become more important over the course of adolescence, especially for girls [11], and there is some evidence that partners assume a more prominent role in early adulthood, particularly for men [14].

Although a developmental pattern in the influences on help-seeking over the course of adolescence and into young adulthood that shows a declining influence of family, increasing influence of friends and growing capacity for self-referral, is conceptually sound and commonly accepted, there is almost no empirical research to confirm this [15]. There are no large-scale studies showing how help-seeking influences change developmentally or whether there are different trends for males and females.

Further, research has yet to investigate the influences of seeking mental health support online. Online mental health care is growing rapidly, and is an increasingly viable alternative to encourage young people to seek help. There is an innovative ‘e-spectrum’ of interventions available to support young people’s mental health and well-being in ways that are congruent with how they live their technology-enhanced lives [16]. Online interventions can have significant advantages for access to mental health care, as they can overcome many of the barriers to seeking help, particularly related to fears about confidentiality, anonymity, self-reliance and stigma. Online support also has the capacity to substantially increase access to mental health interventions by overcoming structural barriers such as cost and availability [17].

Understanding the social influences on seeking mental health care is critical to determine effective pathways to care and how to best support early help-seeking by young people in age and gender-appropriate ways. The current study aimed to investigate the social influences on seeking mental health care for both in-person and online environments for adolescents and young adults, by examining influences on access to a large-scale national Australian network of youth-specific mental health services.

### Hypotheses

The effects of different social influences on youth mental health service use were examined according to type of service delivery modality, gender and age. It was anticipated that the role of family would be most important for younger adolescents; that the influence of friends would peak in mid to late-adolescence; and that self-referral would predominate for young adults. Whether similar patterns were evident for males and females was also explored, along with differences between in-person and online modalities. It was tentatively hypothesised that self and peer referral would be more relevant online due to the high level of autonomy that is inherent online as well as the potential impact of social media, whereas parental influence was expected to be stronger for in-person service access.

## Methods

### Participants

Participants were young people aged 12–25 years seeking help from **headspace** services across Australia. **headspace** is the Australian Government’s National Youth Mental Health Foundation, which commenced in 2006 to respond to the need for early intervention in youth mental health [18]. **headspace** centres have progressively been set up across Australia as an enhanced primary care platform for youth mental health [19]. Centres are free or low-cost youth-friendly primary care service hubs with links to local community and specialist services. At the time of data analysis, there were 55 centres fully operational across Australia; the network is scaling up to 100 centres in 2016, although this will still not provide national coverage in such a large and geographically dispersed country as Australia. A nationally available online service, **eheadspace**, was introduced in June 2011. This is a youth-friendly, confidential, and free mental health support service for young people, with online support provided through webchat, email and via phone, if necessary, although the phone option is the least used. All services are provided by fully qualified and registered mental health professionals.

At both **headspace** centres and **eheadspace**, young people can self-refer, be referred by family or friends, or have a referral from a health or community service provider. The services have been deliberately set up to reduce common barriers to young people accessing mental health care, and consequently, the role of informal referral influence is maximised.

Results reported here are from 30,839 young people who accessed for the first time one of the 55 **headspace** centres fully operational between April 2013 and March 2014, and 7,155 new **eheadspace** clients across the 2013 calendar year. Participants were those who accessed the services initially for mental health or situational concerns. The data come from routine administrative data collection processes, so the results represent a census of clients.

Participating **headspace** centre clients comprised 31.9% males and 54.2% females (13.9% other or not indicated) and **eheadspace** clients were 17.4% males and 80.8% females (1.8% other or not indicated). This gender difference with more female clients accessing online is typical of current online service delivery [20]. The average age of clients was 17.7 (SD = 3.3) and 18.0 (SD = 3.3) for **headspace** centres and **eheadspace**, respectively.

Young people can present to **headspace** services for any type of mental health problem, and access early in the development of mental health problems is promoted and encouraged [8]. Primary issue at presentation is recorded by clinicians through the MDS process (explained below). Participants primarily accessed **headspace** centres for depressive (32.8%) or anxiety (26.0%) symptoms, followed by anger issues (6.9%) and stress-related concerns (5.8%). Depressive (34.5%) and anxiety (17.0%) symptoms were also the most prevalent issues for young people accessing **eheadspace**, followed by difficulty with personal relationships (11.8%) and suicidal thoughts or behaviours (7.9%). Overall, about half the participants had sought prior mental health care and this proportion increased with age: for females by ascending age group, respectively, this comprised 43.8%, 52.9%, 65.5% and 68.5%; and for males was 47.2%, 50.1% 59.4% and 62.5%.

### Procedure

All **headspace** services collect a minimum data set (MDS) of information from all clients who agree to participate, which comprises the vast majority of clients. **headspace** centre clients are asked to fill in MDS information when they first present to the service and at subsequent service occasions. Young people are provided either an iPad or given access to a private computer on which to enter their data. A purpose-built, youth-friendly electronic form has been developed to routinely collect this information. When young people register online for **eheadspace**, they provide basic demographic information and then are sent a confirmation email to activate their account to enable them to use the service. The first time they log-in, they are presented with items from the MDS. Service providers also complete relevant information for each occasion of service through an online form. Data are encrypted to ensure confidentiality and stored in a data warehouse.

Ethics approval was obtained through quality assurance processes, comprising initial consideration and approval by the **headspace** Clinical, Research and Evaluation Committee, and subsequent consideration and approval by the **headspace** Board of Directors. The consent processes were reviewed and endorsed by an independent body, the Australasian Human Research Ethics Consultancy Services. Follow-up data collection processes have been considered and approved by the Melbourne Health Quality Assurance ethics approval.

The measures examined in this study include the basic demographic characteristics of age group (early adolescence: 12–14 years; mid adolescence: 15–17 years; late adolescence: 18–20 years; early adulthood: 21–25 years) and gender (male, female), and responses to the question asked when young people first present to the service, which was “Who most strongly influenced you to come to **headspace** today?” A range of response options is provided (including an open text option, which was coded into appropriate categories), and the frequency of different responses is shown in Table 1.

**Table 1:** **Percentage of young people reporting each main influence by modality, gender and age group**

			Main help-seeking influence						
		*n*	Me	Family	Friend	Partner	Health worker	School staff	Other
**Centres**									
Female	12–14	3036	7.8	57.3	5.7	0.6	12.5	11.3	4.8
	15–17	6339	13.4	40.4	8.3	2.5	16.8	12.4	6.2
	18–20	4035	29.1	23.9	10.3	6.6	19.1	4.1	6.9
	21–25	3316	41.2	15.5	9.8	7.7	18.8	0.9	6.1
Male	12–14	1742	6.0	69.0	3.3	0.6	8.3	8.3	4.5
	15–17	3100	11.6	51.5	5.7	2.9	13.5	8.9	6.0
	18–20	2585	25.5	33.1	7.2	8.0	14.0	3.2	8.9
	21–25	2405	32.4	21.7	8.0	12.5	15.8	0.5	9.1
Total		26558	20.9	39.1	7.3	5.2	14.9	6.2	6.6
**eheadspace**									
Female	12–14	840	64.9	4.8	13.7	0.5	7.3	6.0	2.9
	15–17	2291	69.0	4.3	11.7	1.4	6.8	4.9	2.0
	18–20	1358	73.8	3.4	10.0	3.3	6.3	1.4	1.8
	21–25	1292	81.2	2.2	6.5	4.0	4.1	0.2	1.7
Male	12–14	100	67.4	10.5	9.5	1.1	5.3	5.3	1.1
	15–17	395	65.2	4.0	12.7	3.4	8.2	4.7	1.8
	18–20	345	73.9	2.5	9.9	6.2	6.5	0.6	0.3
	21–25	404	76.7	4.2	9.3	4.0	3.7	0.5	1.6
Total		7025	71.5	4.5	10.4	3.0	6.0	3.0	1.7

### Data analysis

Data were analysed using IBM SPSS v21. Cross-tabulations were used to determine the percentage of young people reporting each help-seeking influence according to modality, gender and age group. A multinomial logistic regression was undertaken to examine whether the factors of modality, gender and age group were associated with help-seeking influence. Because most of the participants had sought prior mental health care, this factor was added as a covariate in the analysis, although it was not shown to make a significant difference to the model when included. Note that a multi-level analysis approach could have been applied to these data, as the young people attending centres are clustered within the higher level unit of the different centres that they attended [21]. Preliminary analyses, however, revealed no clear trends at the higher level unit of the 55 centres and, as this factor was not relevant to the current hypotheses, this analysis approach was not undertaken.

To reveal the different help-seeking patterns in the data, simple effects were examined. With such a complex model, there were many potential effects, but only those of conceptual interest were considered. This comprised the effects of modality, gender and age group on each of the main categories of help-seeking influence: me, family, friend, partner and health worker. Given the very high power due to the large size, significance tests are not reported as negligible effects attain significance. Rather, odds ratios for the strongest effects are presented to aid interpretation of the trends evident in the figures presented.

## Results

Table 1 presents the percentage of young people reporting each main help-seeking influence. It reveals the very large sample size in each group, except for the 12–14 year old boys accessing **eheadspace**.

The multinomial logistic regression showed that the highest level 3-way interaction was significant and a backward stepwise procedure did not eliminate any of the effects. The full factorial model was the best fit, *χ*^2^_(90)_ = 13362.04, *p* < .001, and the Nagelkerke pseudo R^2^ estimated that 34% of the variance in main help-seeking influence was accounted for by the factors of gender, age, modality and their interactions.

**Figure 1:**
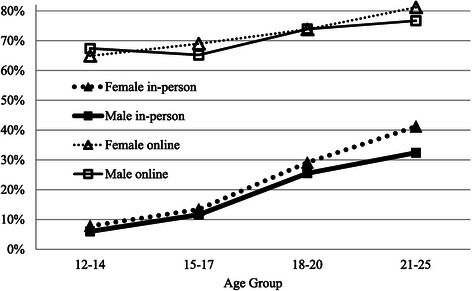
**Percentage who report main help-seeking influence as ‘Me’ by modality, gender and age group.**

Figure 1 graphs the percentage of young people who reported themself as their strongest help-seeking influence. It reveals a strong main effect of modality, with an overall odds ratio showing that self-influence was 10.9 times stronger online than in-person. It also shows a linear increase with age that was much more pronounced for in-person service use and somewhat stronger for females than males. Overall, the young adults were 5.5 times more likely to report themselves as the strongest influence than the early adolescents, however, this varied significantly between modality, being much stronger at 7.9 times for in-person mental health care compared with 2.2 times for online service use.

**Figure 2:**
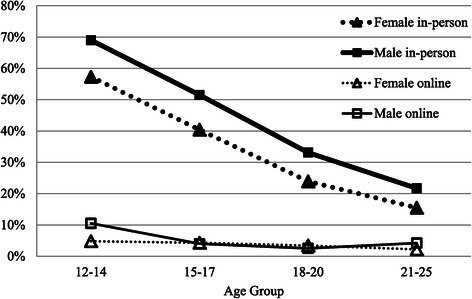
**Percentage who report main help-seeking influence as family by modality gender and age group.**

Figure 2 graphs the percentage of young people who reported family as the strongest help-seeking influence. It reveals a strong main effect of modality, with family influence 15.2 times stronger in-person than online. It also shows a linear decrease with age that was equivalent for males and females accessing in-person mental health care. For in-person services, the early adolescents were 7.6 times more likely to report family as their main influence compared with the young adults. Further, the effect of family for in-person services was stronger overall for males who were 1.6 times more likely to report family influence than females. The influence of family was very low for online support and, consequently, showed less variation by gender and age group.

**Figure 3:**
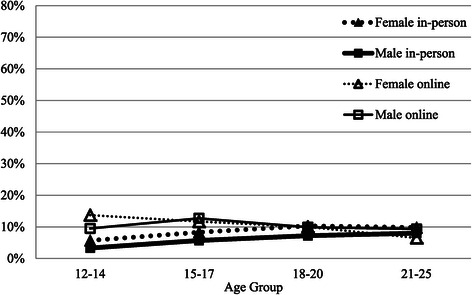
**Percentage who report main help-seeking influence as friend by modality, gender and age group.**

Figure 3 graphs the percentage of young people who reported friends as the strongest help-seeking influence. Overall, the influence of friends was low, being generally less than 10%, with a slightly stronger effect for online help-seeking in early and mid adolescence, where the impact of friends was 1.8 times greater than for in-person services. Overall, in the online environment, friends were 1.3 times more likely to be an influence than in-person. The influence of friends seems to decline with age for online help-seeking, but increase very slightly over the teenage years for in-person mental health service use.

**Figure 4:**
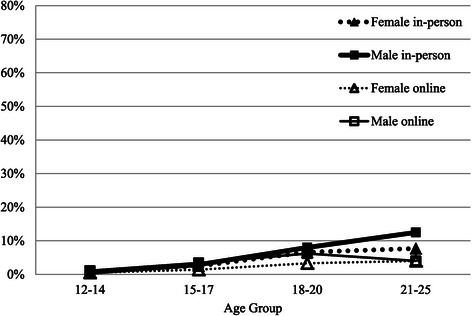
**Percentage who report main help-seeking influence as partner by modality, gender and age group.**

Figure 4 graphs the percentage of young people who reported their intimate partner as the strongest help-seeking influence. Not surprisingly, this effect was almost nil for the younger age groups, but increased with age particularly for males for in-person mental health service use, although this influence remained low. Young adult males were 1.7 times more likely to report their partner as the main help-seeking influence to attend an in-person service compared with young women.

**Figure 5:**
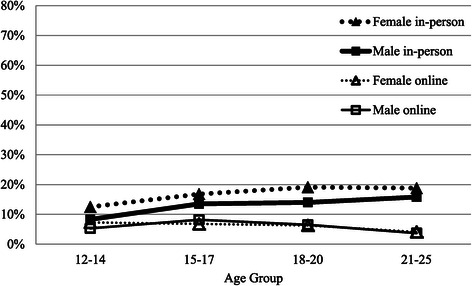
**Percentage who report main help-seeking influence as health worker, by modality, gender and age group.**

Finally, Figure 5 graphs the percentage of young people who reported a health care worker as the strongest help-seeking influence, showing an increasing impact for in-person mental health care, but declining effect with age online. The influence of health care workers became the second strongest, after self, for young women accessing in-person services. Their influence for online service use, however, remained weak.

## Discussion

The aim of this study was to provide the first comprehensive empirical evidence of developmental changes in the social influences on seeking mental health care, both in-person and online, during the vulnerable lifestages of adolescence and young adulthood. Overall, the results show a major developmental shift in help-seeking influences across this age range, and a striking difference between the online and in-person service modalities.

The overwhelming influence of self-initiation of help-seeking online compared with in-person services was clearly evident. The vast majority of both males and females across all age groups self-initiated use of the online service. This was evident even for the younger age groups, with two-thirds of the early adolescent age group reporting that they themselves were the main influence to use **eheadspace**. This is not surprising, although it has previously not been demonstrated through empirical data such as these. A recent Mission Australia report showed that the internet is now the main source of information for young people [22], including health and mental health information [23]. It seems that by exploring and searching online, young people find their own way to online services, without being directed by others.

In contrast, for seeking help in-person, self-initiation was minimal for early adolescents and increased in a linear manner with growing maturity and independence. As developmentally expected, the opposite effect of family was evident, with family being the predominant influence for early adolescents but showing a strong linear decline with age. Nevertheless, even in early adulthood, only 41% of young women and about one-third of young men reported that their strongest help-seeking influence was themselves; and almost a quarter of young adult men still reported their family as the major influence. Family is a stronger influence for males than females for all age groups, which is important to note especially given the larger mental health treatment gap evident for males than females [7].

These findings confirm that a dedicated focus on the factors that affect the help-seeking influence of family is essential to enable young people, particularly males, to access services. A recent systematic review of family and parent factors associated with service use for young people with mental health problems revealed that parental burden, parental problem perception and parental perception of need were most strongly associated with service use [24]. This shows that the mental health literacy of parents, particularly helping parents to recognise mental health problems and be psychologically available to their children with mental health needs, are important targets for interventions to increase service uptake.

The influence of friends was not as strong as might be expected at these lifestages, and was not consistent across age, gender or modality, although there was a weak trend supporting the hypothesis that the influence of friends would be stronger in adolescence, as this influence does drop off somewhat in early adulthood. Interestingly, friend influence appeared to be strongest for the youngest girls within the online environment, and then declined with age. For boys, it peaked online in mid adolescence and then declined. For in-person services, increases for both males and females were evident with age until early adulthood. The stronger influence of friends online for adolescents may be due to the ubiquity of social media and consequent online sharing of websites, stories and interesting information [25]. Youth mental health organisations, such as **headspace**, invest significant effort in their online presence through social media and youth-targeted web material to encourage online sharing, and this may be particularly effective in the early and mid-teen years, as young people increasingly spend a great deal of time online. It may also, however, reveal a cohort effect, with the younger age groups more immersed in the online social environment.

An interesting trend was for the effect of partner, which seemed to be especially relevant for young adult men accessing in-person services. This supports one of the few studies in this area, which showed that for men their partner becomes one of the strongest help-seeking and health care influences, replacing the prior influence of family [14]. In the current data, the general trend for males reveals that the dominant influence for young adults remains the family, with a growing influence of intimate partner, whereas for young women the main influence becomes themselves and, secondarily, their health professionals. These patterns suggest different target groups for influencing the mental health care pathways for males and females.

The role of other ‘gatekeepers’ (shown in Table 1) was overall quite small. Not surprisingly, there was an influence of school staff for school-aged young people, particularly for in-person services, but other community-based gatekeepers were largely irrelevant with older age. This shows that there is considerable potential to increase the impact of such people in the community, and have a greater community-based response to encouraging young people to access appropriate mental health care [26,27].

The findings of the current study have highly relevant implications for help-seeking interventions and policy related to youth mental health. They firstly emphasise that the 12–25 year age range, comprising what in Australia is defined as young people, cannot be treated as a homogeneous group. Developmentally, there is substantial change over this period with little autonomy evident in early adolescence, which grows substantially by young adulthood. Interventions need to be targeted to be age appropriate and recognise the major shift in self-reliance over this age span.

The online environment is clearly distinct from traditional in-person mental health care, and this is the environment that young people are navigating on their own from an early age. Greater self-reliance online, with a slightly stronger peer influence, may be cause for concern, as young people and their friends may not be the best guides to appropriate mental health care. However, with this understanding, the online environment can be tailored to better match young people’s behaviour and meet their needs. Ensuring that online searches for mental health information lead to high quality and evidence-based information and services is essential [28]. Current work in Australia is supporting online mental health organisations to collaborate to develop clear pathways through endorsed online resources that comprise self-help, guided self-help, peer support and fully qualified mental health care (see yawcrc.com.au). They are also engaging social media organisations, like Facebook, to collaborate and accept responsibility to help identify young people at risk and ensure they get to the right types of support online.

There are a number of limitations that must be kept in mind when drawing conclusions from these data. Firstly, it is possible that these findings do not generalise beyond Australia’s health care system, and may be affected by Australia’s significant investment in youth-targeted mental health services through the **headspace** initiative [8]. The results also have not been analyzed according to different cultural backgrounds and there are likely to be important cultural differences in help-seeking influences that have not been revealed. It is likely, however, that cultural factors are more evident for in-person mental health care due to the greater role of family. Another advantage of online mental health support for young people may be that they can overcome cultural, as well as structural, barriers to support.

## Conclusions

These are the first data to show developmental changes across adolescence and young adulthood in the social influences on seeking mental health care, revealing major differences between the in-person and online environments. The results confirm expected developmental trends for in-person service use and provide the first insights into help-seeking influences in the online service environment. Although there is a developmentally appropriate trend toward great self-reliance in seeking help for both modalities, for in-person services the personal connection with parents and family remains paramount, and needs to be supported through family mental health literacy. In the online world, in which today’s young people are constantly immersed, their growing technological sophistication needs to be harnessed to ensure that self-motivated and peer-influenced searching leads to appropriate mental health care pathways.
